# Olfactory Evaluation in Alzheimer’s Disease Model Mice

**DOI:** 10.3390/brainsci12050607

**Published:** 2022-05-06

**Authors:** Jingjing Zhang, Zixuan Zhao, Siqi Sun, Jing Li, Yu Wang, Jingyin Dong, Su Yang, Yiyi Lou, Jing Yang, Weiyun Li, Shanshan Li

**Affiliations:** 1 Department of Basic Medicine, School of Medicine, Zhejiang University City College, Hangzhou 310015, China; hata6r@163.com (J.Z.); zzx001110@163.com (Z.Z.); ssq44747@163.com (S.S.); dongjy@zucc.edu.cn (J.D.); 2 Institute of Neuroscience and Anatomy, School of Medicine, Zhejiang University, Hangzhou 310058, China; lijing851@zju.edu.cn; 3 Hangzhou TCM Hospital Affiliated to Zhejiang Chinese Medical University, Hangzhou 310053, China; wangyu199054@163.com; 4 Sir Run Run Shaw Hospital, School of Medicine, Zhejiang University, Hangzhou 310020, China; 21518362@zju.edu.cn (S.Y.); louyiyi12@163.com (Y.L.)

**Keywords:** olfactory evaluation, Alzheimer’s disease model mice, olfactory behavior test

## Abstract

Olfactory dysfunction is considered a pre-cognitive biomarker of Alzheimer’s disease (AD). Because the olfactory system is highly conserved across species, mouse models corresponding to various AD etiologies have been bred and used in numerous studies on olfactory disorders. The olfactory behavior test is a method required for early olfactory dysfunction detection in AD model mice. Here, we review the olfactory evaluation of AD model mice, focusing on traditional olfactory detection methods, olfactory behavior involving the olfactory cortex, and the results of olfactory behavior in AD model mice, aiming to provide some inspiration for further development of olfactory detection methods in AD model mice.

## 1. Introduction

Alzheimer’s disease (AD) is a slowly progressing disease that remains dormant in the preclinical stage for more than ten years. Early research on the pathological mechanisms of AD primarily focused on irreversible brain damage [1]. Although most clinical symptoms are linked to cognitive impairment, other cognition-related factors should also be considered. Several studies have revealed a direct link between smell, learning, and memory [2,3]. Numerous studies also have found that AD patients experience olfactory dysfunction in the early stages, such as decreased odor discrimination ability, increased olfactory threshold, and olfactory memory loss [4,5,6,7,8,9]. Olfactory detection has been a means of early diagnosis [10,11].

Olfaction is one of the oldest primary sensory systems (i.e., vision, olfaction, taste, hearing, and balance) [12]. The nature of odor receptor proteins, perireceptor processes, the organization of the olfactory central nervous system, and odor-guided behavior and memory are all highly conserved across species [13]. Thus, AD mouse models corresponding to different etiologies of AD have been bred to conduct many studies on AD anosmia, including APP/PS1, Tg2576, 5 × FAD, 3 × Tg, P301S, andApoE4 mice. The olfactory function test is an indispensable research method for verifying AD-like anosmia. This review summarizes commonly used behavioral methods for olfactory detection, the role of the olfactory cortex in olfactory behavior, and the results of olfactory behavior in AD model mice.

## 2. The Structure of the Olfactory System

Primary olfactory areas (the nasal cavity and olfactory epithelium), secondary olfactory areas (the olfactory bulb and lateral olfactory tract), and primary olfactory cortex (the anterior olfactory nucleus, olfactory tubercle, piriform cortex, amygdala, and entorhinal cortex) comprise the olfactory system, which is a component of the sensory nervous system [12,14,15]. Olfactory signals are transmitted step by step in the olfactory system, and volatile odorants reach the olfactory epithelium via the nasal cavity or nasopharynx. Odorant information is converted from chemical signals to neuro-electrical signals after being recognized explicitly by olfactory sensory neurons (OSNs) in the olfactory epithelium and then projected to the main olfactory bulb in a highly precise manner by OSN axons. OSNs expressing the same odorant receptors project to one or more olfactory glomeruli on the surface of the olfactory bulb, forming synaptic connections with mitral cells and tuft cells (M/T) in the olfactory bulb to complete the odor signal handover [16,17]. M/T axons from the lateral olfactory tract further form the anterior olfactory nucleus with medium-sized neurons scattered along the olfactory tract, which eventually branch out at the olfactory tubercle to the fornix (medial striatum) or the piriform cortex, the medial olfactory cortex, and the amygdala (lateral striatum) [15].

## 3. Olfactory Behavioral Tests in Mice

The design of olfactory behavior tests in mice is based on spontaneous drinking or foraging, innate odor memory, and curiosity for novel odors. Integrating all behavioral tests, this review classifies them into three categories: odor recognition tests, odor discrimination tests, and odor memory tests.

### 3.1. Odor Recognition Tests

#### 3.1.1. Food-Seeking Test

**Figure 1:**
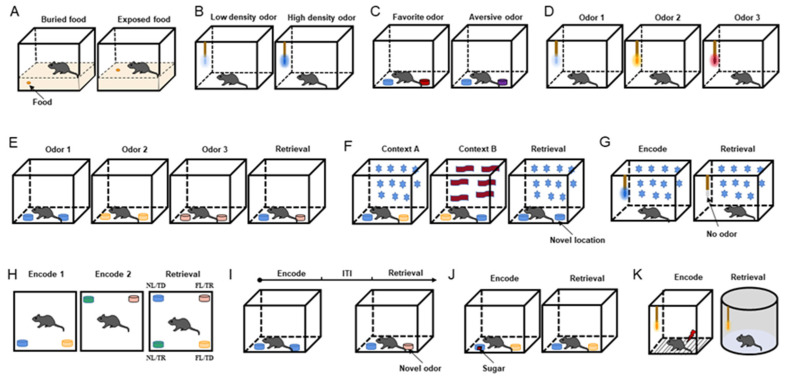
Behavioral paradigms. (**A**) Diagram of food-seeking test. (**B**) Diagram of odor sensitivity test. (**C**) Diagram of odor preference test. Blue is neutral, red is preferred, and purple is aversive. (**D**) Diagram of odor discrimination test. (**E**) Diagram of temporal odor memory test. (**F**) Diagram of spatial odor memory test. Star and wave patterns represent different context decorations. (**G**) Diagram of context odor memory test. (**H**) Diagram of spatiotemporal odor memory test. (**I**) Diagram of context-independent odor memory test. (**J**) Diagram of odor–reward associative memory test. The red square represents sugar cube. (**K**) Diagram of odor–aversion (foot shock) associative memory test. Odorant was applied to the cotton tip (**B**,**D**,**G**,**K**) or odor cup (**C**,**E**,**F**,**H**–**J**). Different colors of cotton swabs or smell cups represent the presentation of different odors.

The food-seeking test is performed to assess general olfactory ability. The mice are trained to look for buried and exposed food with volatile odors (Figure 1A) [18,19,20,21]. To make mice more motivated to seek food, the usual method is to restrict or deprive the mice of food before the experiment and then allow the mice to find the food buried in the bedding and record their latency to find the food. In addition, under the same conditions, an exposed food-seeking test is performed, and the results are compared with those obtained from the buried food-seeking test to confirm olfactory dysfunction without cognitive impairment.

#### 3.1.2. Odor Sensitivity Test

The odor sensitivity test was designed to evaluate the olfactory threshold in mice (Figure 1B) [22,23]. In this experiment, neutral odorants are diluted to various concentrations, such as 1/100, 1/1000, 1/10,000, and 1/100,000, and then presented in increasing order of odor concentration. When the concentration of the presented odor reaches the olfactory threshold, its odor-sniffing behavior increases. In odor concentration presentation, the olfactory threshold experiment outperforms the food-seeking test in terms of controllability and precision.

### 3.2. Odor Discrimination Tests

Odor discrimination tests are classified into two types on the basis of their degree of difficulty: simple and fine. The former selects odors with a significant difference, whereas the latter chooses odors that are similar or contain a mixture of various odors to increase the difficulty of odor discrimination.

#### 3.2.1. Simple Odor Discrimination Tests

##### Odor Preference Test

According to the degree of the innate preference of mice, odors can be classified into three types: innate preferred odor, neutral odor, and innate aversive odor. Researchers designed an odor preference test on the basis of how the mice perceived the three types of odors [20,23]. During the experiment, mice receive two odor combinations—the preferred odor and water and the aversive odor and water—and the sniffing time for each odor is recorded. When the exploring time for the preferred odor is longer than that of water and the exploring time for the aversive odor is shorter than that of water, the mice are considered to have normal discrimination ability (Figure 1C).

##### Neutral Odor Discrimination Test

The neutral odor discrimination test is based on the nature of mice to explore novel odors. The mice are first tested with a non-odorant liquid (mineral oil or water) as a blank odor and then presented with 2–8 odors in sequence to observe the mice’s sniffing behavior for novel odors (Figure 1D). Sniffing for every odor is extremely high in mice with a good sense of smell [24].

##### Odor Cross-Habituation Test

The odor cross-habituation test is employed to evaluate odor habituation and the ability to distinguish between old and new odors (odor dishabituation) [20,21,23]. When mice with intact olfactory function are repeatedly exposed to the same odor, their olfactory exploratory behavior is significantly reduced during the second to fourth exposures, and when a new odor is introduced, they exhibit more sniffing behavior. In general, 3–5 neutral odors are chosen for the experiment, and each odor is presented to the mice 3–4 times consecutively, with a time of 20 s for a single presentation and an interval of 30 s. The difference between the sniffing times for the first and last presentations of each odor represented the degree of habituation, and the difference between the last sniffing time of the previous odor and the first sniffing time of the subsequent new odor represented the degree of dishabituation.

#### 3.2.2. Fine Odor Discrimination Test

The fine odor discrimination test is a more challenging task for mice. Its difference from simple odor discrimination tests is mainly reflected in odor selection. To test the mice’s olfactory fine resolution ability, odors with similar aromas or mixtures of different odors are chosen [23,25,26].

### 3.3. Odor Memory Tests

Context-independent odor memory, context-dependent episodic odor memory, and odor-emotion memory are three types of odor memory tests linked with different olfactory associative cues.

#### 3.3.1. Episodic Odor Memory Tests

##### Temporal Odor Memory Test

This behavioral paradigm was performed to detect the odor temporal order in specific contexts (Figure 1E) [27]. This test included three acquisition phases and one retrieval phase. Different odors were presented in each acquisition stage, and the first odor and the last odor were selected for testing in the retrieval phase with a constant odor spatial context. Correct memory expression prompts the mice to sniff the first odor.

##### Spatial Odor Memory Test

Mice were tested for memory of odor location in a specific context in this paradigm (Figure 1F) [27]. The test chamber was decorated to create two different contexts. Two distinct odors were placed at opposite ends of the chamber in context A (odor 1 on the left and odor 2 on the right). Both odors were placed in opposing positions in context B (odor 2 on the left and odor 1 on the right). The chamber was reconfigured as context A for the retrieval phase, but this time, two copies of odor 1 were presented. The time spent investigating the odor cups (the familiar odor in the old and novel positions) was recorded.

##### Context Odor Memory Test

The context odor memory test was utilized to detect context-driven odor memory (Figure 1G) [27]. In this paradigm, the mice were trained for 30 min on the first nine days to associate a specific environment with an odor presented via a cotton swab, and on the tenth day, the mice were placed in the same context but without the odor. On days 9 and 10, their odor-sniffing behavior was recorded during the first 5 min of their exposure to the context. When the mice failed to detect the expected odor, they used context odor memory by spending more time investigating the swab.

##### Spatiotemporal Odor Memory Test

This behavioral paradigm integrated temporal and spatial odor memory tests (Figure 1H) [27]. During the first acquisition phase, the mice investigated two distinct odors located in two adjacent chamber corners. Following that, two new odors were introduced and placed in the other two corners. Two groups of odors were presented during the retrieval phase, and each group exchanged the spatial location of one odor. Finally, the odor presented four spatiotemporal combinations: familiar location/temporally distant (FL/TD), familiar location/temporally recent (FL/TR), novel location/temporally distant (NL/TD), and novel location/temporally recent (NL/TR). Successful odor memory in time and space leads to a preference for investigating the odor with NL/TR combinations and the least probability of investigating the odor with FL/TD combinations.

#### 3.3.2. Context-Independent Odor Memory Test

This behavioral paradigm conducted the acquisition phase and the retrieval phase with the same spatial cues in the same context (Figure 1I) [26]. During the acquisition phase, mice were placed in the chamber with two small cups containing the same odor that were placed on opposite sides of the arena. After exploring both copies, the animal was placed in a holding cage for 5, 15, or 30 min. During the retrieval phase, mice investigated both old and new odors. If mice retained memories of old odors, they were more inclined to explore new odors.

#### 3.3.3. Odor–Emotion Memory Tests

##### Odor–Reward Associative Memory Test

The reward in an odor–reward associative memory test can be food, candy, or water. During the acquisition phase, the mice were exposed to two odors, one with a reward (R+) and one without a reward (R-). Mice were repeatedly trained and successfully associated the R+ odor with the behavior of finding hidden food, candy, or drinking water. During the retrieval phase, the association behavior of the R+ odor matches was recorded (Figure 1J). Mice with a well-established odor–reward combined memory were more likely to dig in the odor cup of the R+ odor or drink water when the R+ odor appeared [23,28,29].

##### Odor–Aversion Associative Memory Test

Aversion can be induced in the odor–aversion associative memory test by using water-containing LiCl to induce stomach upset, causing avoidance behavior, or a shock to startle mice, resulting in freezing behavior (Figure 1K) [30,31,32]. Like the odor–reward combined memory test, it was necessary to successfully associate an odor with an aversive behavior and then observe the corresponding behavior during the odor retrieval phase.

## 4. Olfactory Behavior Involving the Olfactory Cortex

Olfactory behavior is relatively complex, and an olfactory behavior may involve multiple brain regions. Systematic studies on the involvement of all primary olfactory cortices in a given olfactory behavior are lacking. Here, we summarize the current research progress regarding the involvement of the primary olfactory cortex in olfactory behavior (Table 1).

### 4.1. Anterior Olfactory Nucleus

The anterior olfactory nucleus (AON) is located behind the OB, anterior to the piriform cortex and olfactory tubercle. It is divided into four subregions: the medial anterior olfactory nucleus (mAON), the dorsal anterior olfactory nucleus (dAON), the lateral anterior olfactory nucleus (lAON), and the ventral anterior olfactory nucleus (vAON) [33]. Olfactory sensitivity is modulated by the excitatory input of mAON neurons to the OB [34]. The AON’s episodic odor memory is modulated by hippocampus (HPC) input [34,35,36]. Hippocampal projections to the AON are position-specific, i.e., the intermediate HPC (iHPC) innervates the lAON, and the ventral HPC (vHPC) innervates the mAON [37]. The spatial and temporal representations are distributed differently throughout the AON. Although the iHPC–lAON and vHPC–mAON pathways transmit spatial information, only the vHPC–mAON pathway supports temporal information [35].

### 4.2. Olfactory Tubercle

The olfactory tubercle (OT), part of the ventral striatum, connects the limbic and basal ganglia systems to facilitate behavioral learning [38]. Rather than odor identities, specific domains of the OT represent odor-induced distinct motivated behaviors. Mice were trained to associate odor cues with reward or punishment to induce odor-guided approach behavior or aversive behavior. It was found that odor cues that caused approach behaviors activated the anteromedial region of the OT, whereas odor cues that caused aversive behaviors activated the lateral region [30]. As a result, the OT is regarded as a candidate region involved in odor-induced motivational behavior, in which odor valence is encoded to guide goal-directed behavior [30,39,40].

### 4.3. Piriform Cortex

The piriform cortex (PC) is the largest component of the olfactory cortex and has been described as a critical area for odor encoding and memory [41,42,43]. The PC is roughly divided into the anterior piriform cortex (aPC) and the posterior piriform cortex (pPC). The aPC receives more afferent input from the OB and less associative input, whereas the pPC receives the opposite [44,45,46,47]. The aPC encodes odor identity [48] and participates in odor–reward memory [32,49,50], while the pPC encodes odor valence and is involved in odor–aversion memory acquisition [31,47,50]. The PC, conceptualized as the associative cortex, combines incoming olfactory information with descending input from advanced associative areas.

### 4.4. Entorhinal Cortex

The entorhinal cortex is a brain region linked to the hippocampus, amygdala, olfactory bulb, and piriform cortex. Therefore, it is believed to play an essential role in olfactory learning and memory [51]. According to studies, LEC lesions can impair recall of well-learned difficult odor recognition tasks but do not affect well-learned simple odor recognition tasks [48,52]. Furthermore, LEC lesions can impair the retrieval of odor–context associative memory [52] and spatial odor memory [53], while promoting the acquisition of odor–aversive associative memory [54,55].

### 4.5. Amygdala

The amygdala is the central station for acquisition, extinction, and consolidation of emotional memory [56], and it is involved in both innate odor-driven behavior and acquired olfactory behavior [57]. The basolateral amygdala only plays a regulatory role in innate fear behavior [58,59,60,61], but it is essential in social same-sex odor preference behavior, odor–reward associative memory [62], and odor–aversion associative memory [31,63,64,65,66]. The central amygdala is involved in the acquisition and consolidation of odor–aversive associative memory [67]. The medial amygdala plays a key role in innate fear behavior [61,68,69], social heterosexual odor preference [60,61,62,63,64,65,66,67,68,69,70,71,72,73], and episodic odor memory [74]. The cortical amygdala plays a vital role in developing innate odor-driven behaviors (innate attraction and innate fear behavior) [59].

## 5. Behavioral Performance of AD Model Mice

Amyloid plaques caused by an amyloid precursor protein (APP) gene mutation, neurofibrillary tangles (NFT) caused by tau protein gene mutation, missense mutations of the presenilin-1 (PS-1) gene, and the apolipoprotein E typeE4 (ApoE4) allele are closely associated with Alzheimer’s disease [75,76,77,78]. Here, we summarize the behavioral results of AD model mice with related gene mutations.

APP/PS1 mice co-express a chimeric mouse/human mutant APP gene (Swedish mutations K594N and M595L) and human PS1 encoding an E9 deletion [79]. The amyloid burden in the olfactory system began to occur in APP/PS1 mice at 3–4 months of age and became more pronounced with increasing age, resulting in neuronal atrophy, synaptic loss, axonal degeneration, neuroinflammation, and decreased neurogenesis [25,80,81,82,83,84,85,86]. Phosphorylated tau appearance occurred much later than amyloid deposition [87]. In addition, female APP/PS1 mice developed amyloid deposition earlier than male mice [88]. In the buried food test, starting from the age of three months, APP/PS1 mice took longer to find the hidden food than age-matched wild-type (WT) mice. In the olfactory sensitivity test, when the lowest odor concentration was presented at a ratio of 1 to 10,000, neither the 3-month-old nor the six-month-old APP/PS1 mice showed obvious abnormalities compared with age-matched WT mice [89]. Furthermore, 8- and 9-month-old APP/PS1 mice spent less time sniffing attractive odors and more time exploring aversive odors than age-matched WT mice in an odor preference test. Similarly, there was no significant difference in the time spent by APP/PS1 mice to distinguish between water, attractive odors, and aversive odors [82,90]. In the fine odor discrimination experiment, researchers found that odor habituation was not altered in 12-month-old APP/PS1 mice, but fine discrimination capacity was impaired [25,26]. Furthermore, two-odor discrimination tests revealed that APP/PS1 mice’s ability to discriminate was impaired. In the context-independent odor memory test, WT mice retained the memory of the first odor and explored more novel odors 15 min after the first odor presentation; APP/PS1 mice, on the other hand, only performed the same 5 min after the first odor presentation [26]. Thus, 12-month-old APP/PS1 mice exhibited defects in familiar odor retention (Table 2).

Tg2576 mice overexpress the human APP 695 amino acid isoform with the double Swedish mutation (K670N, M671L) [92]. The olfactory bulb is the earliest brain region to deposit Aβ, and the spatial–temporal pattern of Aβ deposition in the olfactory bulb is correlated with olfactory deficits [93]. Apoptosis, neurotransmitter disturbance, and neuroinflammation induced by Aβ were observed in aged Tg2576 mice [94,95,96,97]. The odor cross-habituation test [98], fine odor discrimination test [99], and relatively simple odor-associated behavioral tasks (12 odors) [100] revealed no abnormalities in 4-month-old Tg2576 mice. When they performed more challenging odor-associated behavioral tasks (22 odors), they demonstrated disrupted olfactory memory, which was aggravated with age [100]. Furthermore, 6-month-old Tg2576 mice exhibited the longer latency of seeking buried food [18], altered odor habituation/dishabituation [98,99,101], and odor memory impairments (Table 3) [102].

5xFAD mice harbor both mutant human APP with Swedish (K670N, M671L), Florida (I716V), and London (V717I) mutations and human PS1 harboring two mutations (M146L, L286V) [103]. Aβ accumulates in the glomeruli of the olfactory bulb as early as 1 month of age and increases with age [104]. Similarly, in aged 5xFAD mice, extensive plaque deposition, neuronal loss, and neuroinflammation occur [105,106,107]. Compared with WT mice, 3-month-old 5xFAD mice had significantly longer latency to seek buried food. There were no detection deficits in recognizing one type of odor in 2-, 4-, or 6-month-old 5xFAD mice [40]. However, Girard et al. found that mice exhibited partial impairments in odor detection when the variety of odors recognized increased [24]. At any odor concentrations (vapor concentrations of odorant of 1, 0.1, 0.01, 0.001, 0.0001, and 0.00001 ppm), there was no difference in performance between 6-month-old 5xFAD and WT mice [108]. In the odor–reward associative test, after reducing the number of odor–reward trials in each session and only counting the correct rate of the first trial in each session, it was found that 4-month-old 5xFAD mice developed odor–reward association deficits [29]. These findings suggest that olfactory deficits in 5xFAD mice before 6 months of age are correlated with odor memory rather than simple odor detection. However, olfactory discrimination impairments were observed in 8-month-old 5XFAD mice (Table 4) [109].

Three mutant alleles, the PSEN1 mutation (M146V), the APP Swedish (K670N/M671L), and the tau P301L transgenes, are expressed in 3 × Tg mice [110]. In contrast to pathological deposition in APP/PS1 and Tg2576 mice, Aβ and tau pathology in 3 × Tg mice was absent in the olfactory bulb but present in the piriform cortex, entorhinal cortex, or hippocampus [111]. However, neuron loss and neuroinflammation can also occur. Interestingly, amyloid deposition precedes tau pathology [110]. The 3- to 5-month-old 3 × Tg mice demonstrated increased latency in locating buried food [91], but no difference was shown in 3.25- and 4.5-month-old 3 × Tg mice [112]. The seemingly contradictory results may be due to differences in the sex ratio of experimental animals or differences in neuropsychiatric symptoms in experimental animals [112,113]. Six-month-old 3 × Tg female mice showed decreased olfactory detection at odor vapor concentrations of 0.00001 ppm, while males showed no difference [108]. However, in the odor cross-habituation test, male and female 6-month-old 3 × Tg mice behave normally (Table 5) [114].

P301S mice express the P301S mutant form of human microtubule-associated protein tau [115]. Mitral cells in the olfactory bulb and piriform cortex have been immunolabeled with hyperphosphorylated tau antibody in 1-month-old P301S mice [116]. P301S mice displayed progressive neuronal cell loss in the olfactory bulb and piriform cortex [116]. Impaired gamma oscillations at the OB circuit were detected in 3-month-old P301S mice [117]. Synapse loss, impaired synaptic function, and microglial activation in the hippocampus were prior to tangle formation [118]. Two-month-old P301S mice exhibited increased latency in finding buried food [19]. The olfactory sensitivity and odor cross-habituation was significantly impaired at 3 months of age [116]. Another AD model mouse is the ApoE4 mouse, which is homozygous for the human ApoE ε4 allele (arg112, arg158) [119]. Odor-evoked response magnitudes in ApoE4 mice increase in both the olfactory bulb and piriform cortex [120,121]. Impaired odor habituation was found in ApoE4 young mice at 6 months of age, but no difference was detected at 12 months of age [121] (Table 6). The degree of odor habituation in ApoE4 mice is determined by both genotype and age [120,121].

## 6. Overview

Olfactory dysfunction is proposed to be an early biomarker of AD. The detection of olfactory function as an auxiliary method in the clinical diagnosis of AD patients has received increasing attention. Consequently, a large number of olfactory behavior studies have been applied in AD model mice. According to current experimental results, 2- to 3-month-old AD model mice have an elevated odor threshold and difficulty discriminating between odors, indicating that early olfactory dysfunction in AD mice can closely mimic the clinical manifestations of AD patients. Studies on olfactory function in AD mice have mainly focused on odor threshold detected by odor recognition tests and discrimination ability detected by odor discrimination tests, but they have relatively rarely focused on olfactory-associated memory detected by odor memory tests. The same phenomenon also exists in the detection of olfactory function in AD patients [122,123]. Odor memory loss is also a symptom of AD onset [8,9], but studies on mice have only been conducted in the elderly. To expand the range of applications of AD mice, odor memory tests need to be carried out in AD model mice at a young age. To summarize the behavioral experimental methods, it was found that researchers improved conventional behavioral methods by increasing odor types or decreasing the number of repetitions in the odor memory test training process, thereby increasing the difficulty of odor identification and odor memory acquisition. This provides some reference methods and improvement ideas for future research.

## Figures and Tables

**Table 1:** Olfactory behavioral summary of olfactory cortex involvement.

Behavioral Tests	Anterior Olfactory Nucleus	Olfactory Tubercle	Piriform Cortex	Entorhinal Cortex	Amygdala
Food-seeking test	√				
Odor sensitivity test	√				
Odor preference test					√
Neutral odor discrimination test			√		
Odor cross-habituation test			√		
Fine odor discrimination test			√		
Context-independent odor memory test					
Context odor memory test	√			√	√
Temporal odor memory test	√				
Spatial odor memory test	√			√	
Odor–reward memory test		√			√
Odor–aversion memory test		√		√	√

**Table 2:** Summary of data on the behavioral performance of the APP/PS1 mice; “3–5 Mo-M:F-1:1-X” represents “age-sex-sex ratio-whether there is a difference”. Mo: month, M: male, F: female, N: unclear, ✕: no significant difference, √: significant difference.

APP/PS1 Mice	1–2 Mo	3–5 Mo	6 Mo	8 Mo	9–10 Mo	11–12 Mo
Food-seeking test	M:F-1:1-✕ [25]	3–5 Mo M:F-N-√ [91] 3–4 Mo M:F-1:1-√ [25]	6–7 Mo M:F-1:1-√ [25]	M-√ [90] F-√ [90]	9 Mo M-√ [82] 9–10 Mo F-1:1-√ [25]	11–12 Mo F:M-N-√ [91]
Odor sensitivity test		3 Mo F-✕ [89]	F-✕ [89]			
Odor preference test				M:F-10:7-√ [90]	9 Mo M-√ [82]	
Odor cross-habituation test						12 Mo M:F-N-√ [26]
Fine odor discrimination test		3 Mo M:F-1:1-√ [25]	M:F-1:1-√ [25]		10 Mo M:F-1:1-√ [25]	12 Mo M:F-N-√ [26]
Context-independent odor memory test						12 Mo M:F-N-√ [26]

**Table 3:** Summary of data on the behavioral performance of the Tg2576 mice; “3–4 Mo M:F-1:1-X” represents “age-sex-sex ratio-whether there is a difference”. Mo: month, M: male, F: female, N: unclear, ✕: no significant difference, √: significant difference.

Tg2576 Mice	3–4 Mo	6 Mo	7 Mo	12 Mo	14 Mo	16 Mo	21 Mo
Food- seeking test		M:F-N-√ [18]			sexual N-√ [18]		
Odor cross-habituation test	3–4 Mo M:F-1:1-✕ [93,98]	6–7 Mo M:F-1:1-√ [93,98]		M:F-N-√ [99]		M:F-N-√ [101] M:F-1:1-√ [93]	21–29 Mo M:F-1:1-√ [93]
Fine odor discrimination test	3 Mo M:F-N-✕ [99]	M:F-N-✕ [99]		M:F-N-✕ [99]			
Odor–emotion memory test	4 Mo M-12 odors-✕ [100] 4 Mo M-22 odors-√ [100]	M-√ [102]	F-√ [38] M-12 odors-√ [84] M-22 odors-√ [84]	M-12 odors-√ [100] M-22 odors-√ [100]		F-√ [28]	
Context-independent odor memory test		M-√ [102]					

**Table 4:** Summary of data on the behavioral performance of the 5xFAD mice. “M-X” represents “age-sex-whether there is a difference”. Mo: month, M: male, F: female, ✕: no significant difference, √: significant difference.

5xFAD Mice	2 Mo	3 Mo	4 Mo	6 Mo	8 Mo
Food-seeking test		M-√ [24]			
Odor sensitivity test				M-✕; F-✕ [108]	
Simple odor discrimination test	M-✕ [29]	M-√ [24]	M-✕ [29]	M-✕ [29]	
Odor cross-habituation test			M-✕ [109]		M-√ [109]
Odor–emotion memory test			M-√ [29]	M-√ [29]	

**Table 5:** Summary of data on the behavioral performance of the 3 × Tg mice; “3–5 Mo M:F-1:1-X” represented “age-sex-sex ratio-whether there is a difference”. Mo: month, M: male, F: female, N: unclear, ✕: no significant difference, √: significant difference.

3 × Tg Mice	3 Mo	6 Mo	9.75 Mo	13 Mo
Food-seeking test	3–5 Mo M:F-N-√ [91] 3.25 Mo M:F-1:1-✕ [112] 4.5 Mo M:F-1:1-✕ [112]		M:F-1:1-√ [112]	M-√; F-√ [113]
Odor sensitivity test		M-X; F-√ [108]		
Odor cross-habituation test		M-X; F-✕ [114]		

**Table 6:** Summary of data on the behavioral performance of the P301S and the ApoE4 mice. “M:F-N-X” represented “sex-sex ratio-whether there is a difference”. Mo: month, M: male, F: female, N: unclear, ✕: no significant difference, √: significant difference.

		1 Mo	2 Mo	3 Mo	5 Mo	6 Mo	9 Mo	12 Mo
Food-seeking test	P301S mice		M-√ [19]		M-√ [19]		M-√ [19]	
Odor sensitivity test	P301S mice	M-✕ [116]		M-√ [116]	M-√ [116]			
Odor cross-habituation test	P301S mice			M-√ [116]				
	ApoE4 mice					M:F-N-√ [121]		M:F-N-✕ [121]
